# Fungal Shoulder Periprosthetic Infections: A Systematic Review

**DOI:** 10.3390/jcm13206128

**Published:** 2024-10-14

**Authors:** Vasileios Giovanoulis, Vasileios Pastamentzas, Enejd Veizi, Charalampos Matzaroglou, Symeon Naoum, George Samonis, Maria Piagkou, Dimitrios V. Papadopoulos, Andreas G. Tsantes, Christos Koutserimpas

**Affiliations:** 1 Department of Orthopaedic Surgery, Hôpital Henri Mondor, AP-HP, Université Paris Est Créteil (UPEC), 94010 Creteil, France; vasigiova@gmail.com; 2 Department of Orthopaedics and Traumatology, “251” Hellenic Air Force General Hospital of Athens, 11525 Athina, Greece; v.pastamentzas@gmail.com; 3 Department of Orthopedics and Traumatology, Yıldırım Beyazıt University, Ankara City Hospital, Ankara 2367, Turkey; dr.nad89@hotmail.com; 4 Department of Physiotherapy, School of Health Rehabilitation Sciences, University of Patras, 26504 Rio, Greece; matzaroglou@upatras.gr; 5 Department of Trauma and Orthopaedics, Royal Berkshire Hospital, Reading RG1 5AN, UK; naoumsimeon@gmail.com; 6 School of Medicine, University of Crete, 71003 Heraklion, Greece; samonis@med.uoc.gr; 7 First Department of Medical Oncology, Metropolitan Hospital of Neon Faliron, 18547 Athens, Greece; 8 Department of Anatomy, School of Medicine, Faculty of Health Sciences, National and Kapodistrian University of Athens, 15772 Athens, Greece; piagkoumara@gmail.com; 9 2nd Academic Department of Orthopaedics, School of Medicine, National and Kapodistrian University of Athens, 14233 Athens, Greece; di_papadopoulos@yahoo.gr; 10 Microbiology Department, “Saint Savvas” Oncology Hospital, 11522 Athens, Greece; andreas.tsantes@yahoo.com; 11 Laboratory of Hematology and Blood Bank Unit, “Attikon” University Hospital, School of Medicine, National and Kapodistrian University of Athens, 12462 Athens, Greece; 12 Orthopaedic Surgery and Sports Medicine Department, Croix Rousse, University Hospital of Lyon, 69004 Lyon, France

**Keywords:** arthroplasty, joint infection, arthritis, fungal infection, *Candida*, bone infection, shoulder reconstruction

## Abstract

**Background**: Data regarding fungal PJIs of the shoulder are scarce. The present systematic review aims to identify and evaluate all published shoulder fungal PJIs in an effort to better understand the diagnostic and therapeutic approach to these infections. **Methods**: A systematic review according to the PRISMA guidelines was conducted, locating all shoulder fungal PJIs. The initial search located 1435 articles. Data were collected on demographics, the causative fungus, antifungal treatment (AFT), surgical interventions, and infection outcomes. **Results:** After screening and implementation of the inclusion criteria, a total of 10 articles, including 10 cases, were eligible. The sample’s mean age was 62.44 years. Diabetes mellitus was the most common comorbidity (30%), while 70% were immunocompromised. *Candida* spp. was the most common causative fungus (nine cases; 90%), while all cases were confirmed with cultures. In three cases (30%), there was bacterial co-infection. The mean duration of antifungal treatment (AFT) was 8.4 weeks, while the preferred agent was fluconazole (60% of cases), followed by amphotericin B (30%). Most cases (50%) underwent resection arthroplasty as part of the treatment, while two-stage revision arthroplasty was performed in 30%. Infection’s eradication was reported in 90% of the studied cases. **Conclusions**: The diagnosis and management of fungal periprosthetic shoulder infections are particularly challenging and require a multidisciplinary approach. The combination of antifungal therapy and tailored surgical strategies is crucial, but further research is needed to refine treatment protocols and address the unique considerations in shoulder PJIs.

## 1. Introduction

Shoulder arthroplasty (SA) is a highly effective and commonly executed orthopedic surgery, especially beneficial for patients suffering from a range of painful degenerative disorders of the shoulder joint. SA has shown a five-fold increase during the last decade in the United States of America and a 5.6-fold in the last two decades in the United Kingdom [1,2]. The global SA market was valued at 1.52 billion USD in 2022 and it is estimated to reach 2.60 billion USD by 2030 [3].

There are three primary types of shoulder arthroplasty implants: hemiarthroplasty, anatomic shoulder arthroplasty, and reverse shoulder arthroplasty [4]. Hemiarthroplasty involves replacing only the humeral head and is typically indicated for patients with intact glenoid cartilage but damaged humeral surfaces, such as those with avascular necrosis or certain fractures. Anatomic shoulder arthroplasty, which replicates the natural anatomy by replacing both the humeral head and glenoid, is preferred for patients with intact rotator cuff function and osteoarthritis. In contrast, reverse shoulder arthroplasty as proposed by Grammont is indicated for patients with irreparable rotator cuff tears, cuff tear arthropathy, or complex fractures, as it reverses the normal ball-and-socket configuration to rely on the deltoid muscle for shoulder movement [4,5]. Each type of implant has specific indications, with the choice depending on the underlying pathology, the condition of the rotator cuff, and the patient’s functional needs [5,6,7,8,9,10]. It is important to highlight that many of the reported cohorts do not differentiate between the various types of arthroplasties. Consequently, this lack of distinction prevents accurate reporting of periprosthetic joint infection (PJI) incidence based on arthroplasty type. Nevertheless, with the expanding indications for both total and reverse shoulder arthroplasty, particularly in trauma cases, the incidence of PJI is anticipated to increase. Complication rates after shoulder arthroplasty range from 7% to 25%, with PJIs estimated to occur in 3% to 8% of all-cause cases [1,11]. PJIs represent a major complication in joint reconstruction that may lead to multiple surgical interventions, long-term antimicrobial treatment and increase of in-hospital stay and costs, as well as significant decrease of the patient’s quality of life [11]. These infections can significantly diminish the patient’s life quality and increase comorbidity [12]. Factors that increase the risk of PJI include extended surgery duration, postoperative complications like hematoma and wound dehiscence, the use of a tourniquet, especially in knee arthroplasty, as well as comorbid conditions such as diabetes mellitus (DM), cancer, chronic renal disease, obesity, immunosuppression, and a higher American Society of Anesthesiologists (ASA) score. Additionally, the need for blood transfusion has also been identified as a contributing factor [10,13,14].

In 2018, the International Consensus Meeting on Musculoskeletal Infection established guidelines for diagnosing PJIs after shoulder arthroplasty, categorizing them as definite, probable, possible, or unlikely. A ‘definite’ infection is confirmed by a sinus tract, intra-articular pus, or two identical positive cultures with virulent organisms. For cases not meeting these criteria, a scoring system based on minor criteria, such as unexpected wound drainage, single positive cultures, humeral stem loosening, and elevated inflammatory markers is used [15].

PJIs represent an uncommon complication, affecting approximately 1% of primary total shoulder arthroplasty (TSA) cases, while in revision cases this rises to almost 4% [16]. However, with the increasing number of TSAs being performed, the incidence of PJI is projected to grow. Classic clinical signs of infection are less frequently observed in shoulder PJI, largely due to the prevalence of slow-growing pathogens like *Cutibacterium acnes*. Infections caused by such indolent organisms, such as fungi, or those presenting atypically may lead to delayed diagnosis, potentially impacting the selection of surgical interventions and the overall treatment success [17]. Fungi are very uncommon causes of PJIs, appearing in only approximately 1% to 2% of cases [18]. This rate has risen over recent years due to the older population and the number of immunosuppressed hosts [12,19,20,21]. Currently, there are no official guidelines for treating fungal PJIs affecting the shoulder [18,22]. The purpose of this systematic review is to identify and assess all published cases of fungal PJIs following SA. Specifically, the study seeks to better understand the incidence, diagnostic approach, management, and prognosis of these rare but severe infections and contribute to the development of more effective treatment protocols and improved patient outcomes.

## 2. Materials and Methods

### 2.1. Study Design

This systematic literature review adhered to the PRISMA (Preferred Reporting Items for Systematic Reviews and Meta-Analyses) guidelines and was conducted according to the protocol established and agreed upon by all authors. It was also registered in the International Prospective Register of Systematic Reviews (PROSPERO) with reference number CRD42024571557. A meticulous electronic search of PubMed, MEDLINE, and Scopus databases was carried out to identify all existing articles reporting cases of fungal PJIs of SAs [23]. Articles were searched up to July 2024. The following search algorithm was used: (((Shoulder) OR (“shoulder arthroplasty”)) OR (“shoulder replacement”)) AND ((((Fungal) OR (Fung*)) OR (*Candida*)) OR (*Albicans*)). After identifying these cases, the individual references cited in each publication were further examined to discover additional cases.

### 2.2. Eligibility Criteria

Randomized controlled trials, case series, comparative, observational, and cohort studies, as well as case reports that assessed cases of fungal PJIs of SA (including hemiarthroplasty, anatomic total SA, and reverse SA) in adult patients were included in this review. The review was limited to peer-reviewed journals. Proceedings from scientific meetings were not included. The articles were not limited to any language. 

### 2.3. Study Selection

Two investigators (V.G. and V.P.) independently conducted the literature search. Initially, articles were screened and selected based on their titles and abstracts according to the inclusion criteria. Studies that did not meet the criteria were excluded. The full texts of the included papers were then reviewed. Any disagreements between the two reviewers were resolved through discussion and consensus with a third investigator (C.K.). A list of excluded studies and the reasons for their exclusion were noted.

### 2.4. Data Extraction and Analysis

The same authors (V.G., V.P.) independently reviewed the articles and extracted the data for each study in a predefined Microsoft Excel spreadsheet (Microsoft Corporation, Redmond, WA, USA). The extracted data encompassed age, gender, country of origin, the presence of immunosuppressive conditions/comorbidities, the indication for the initial SA, the time period between initial prosthesis implantation and symptoms’ onset, as well as the time period between symptoms’ onset and firm diagnosis and between symptoms’ onset and the previous use of antimicrobials within the previous 12 months. Furthermore, the presence of bacterial co-infection, mechanism of infection, clinical manifestation, microbiological, laboratory, and imaging findings, number of previous surgeries in the same joint, duration and type of AFT, as well as the type of surgical intervention were recorded and evaluated. Moreover, the outcome of medical and surgical treatment, along with the follow-up of each case (after treatment initiation for the PJI), were studied. Treatment was deemed successful when all signs and symptoms of the infection had resolved and no recurrence was detected throughout the follow-up period. Any discrepancies were resolved through consensus with the senior author (C.K.). The risk of bias was assessed using the bias risk tool in case reports and series developed by Murad et al. [24].

## 3. Results

**Figure 1:**
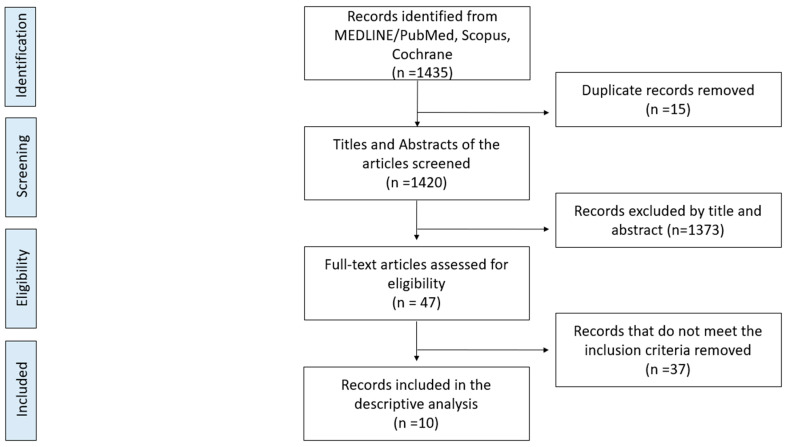
Preferred Reporting Items for Systematic Reviews and Meta-Analyses (PRISMA) flowchart for this study.

Of the initial 1435 articles identified through the search strategy, a total of 47 articles remained after the abstracts were screened for eligibility. After full-text evaluation, review of the references from each included article, and application of the inclusion–exclusion criteria, a total of 10 articles were eligible for this systematic review [25,26,27,28,29,30,31,32,33,34]. A flowchart of the literature search is exhibited in Figure 1. All included studies (100%) were either single cases from case reports (seven papers [25,27,28,29,30,31,32]) or single cases as part of small series (three papers [26,33,34]).

The risk-of-bias assessment, conducted using the bias risk tool for small, nonrandomized studies, indicated that two studies (20%) [26,33]) were found to have a low overall risk of bias, while eight studies (80%) [25,27,28,29,30,31,32,34]) had moderate risk. This was primarily due to the fact that many of the case series and case reports did not capture the complete experience of the investigator [24] (Table 1).

In total, 10 patients (four males [27,28,29,31], five females [25,26,30,32,34]; gender was not reported in one case [33]) were included in the study. The age of patients ranged from 40 to 71 years with a mean value 62.44 [standard deviation (SD) = 8.98]. The type of arthroplasty varied among cases, including four cases (40%) of anatomic total shoulder arthroplasty [26,29,31,34], three (30%) of reverse shoulder arthroplasty [27,28,30], as well as two (20%) of hemiarthroplasty [25,32], while the arthroplasty type was not reported in one case [33]. The body mass index (BMI) was reported in two cases with mean value 30.6 kg/m^2^ (SD = 0.7) [26,27]. The mean follow-up of these cases was 22.2 months (SD = 13.33).

The primary indication of SA before the development of fungal PJI was in most cases (five cases; 50%) traumatic due to fractures [26,27,28,29,32], followed by two cases of osteoarthritis (20%) [31,34] and two cases of rheumatoid arthritis (20%) [25,30], while in one case (10%) [33]), the reason was not reported. Two cases had a history of previous surgery of the shoulder joint (two previous interventions in one case [26], and one in the other [27]).

Regarding the causative fungus, *Candida parapsilosis* was yielded in four cases (40% [28,29,33,34]) and *Candida albicans* in three cases (30%) [25,30,32]). Furthermore, a case (10% [27]) of *Candida glabrata* and another (10% [31]) of *Cryptococcus magnus* were also identified. In addition, there was another *Candida* species in one patient that was not further characterized [26]. In all cases, microbiological examination revealed the causative fungus. Tissue samples were the most commonly used cultured material (six cases; 60% [26,28,30,31,32,33]), followed by joint fluid aspiration (four cases; 40% [25,27,29,34]). Furthermore, in three cases (30%), a concomitant bacterial co-infection was reported: a methicillin-resistant *Staphylococcus epidermidis* (MRSE) [31], a *Serratia marcescens* [27], and *Staphylococcus* species [34] (Table 2).

Diabetes mellitus (DM) was the most common comorbidity among the included patients (three cases; 30% [25,27,31]), followed by rheumatoid arthritis (two cases; 20% [25,30]). Notably, seven patients (70% [25,26,27,29,30,31,34]) could be immunocompromised according to their comorbidities, and five patients (50% [25,26,31,32,34]) had a history of antibiotic use within the previous 12 months. History of previous surgery in the infected joint was noted in two patients (20% [27,29]). Both of them underwent open reduction and internal fixation of a shoulder fracture.

The infection was spread hematogenously in three instances (30% [25,30,31]), followed by direct inoculation in one (10% [28]), while in the remaining six cases the infection’s spread was not specified. Regarding the presenting signs and symptoms, local infection signs such as swelling were reported in most cases (six cases; 60% [25,28,29,30,32,34]), followed by persistent pain (five cases; 50% [25,27,28,32,34]) and elevated local temperature (three cases; 30% [24,27,29]). Moreover, three patients (30% [25,29,30]) suffered decreased range of motion of the affected shoulder joint, two patients had erythema on the shoulder (20%) [30,34], and one (10% [27]) experienced shoulder instability. Systematic symptoms including pyrexia were reported in two patients (20% [28,32]). The average duration from the initial implantation to the onset of symptoms was 21.53 months (SD = 26.73), whereas the average time from symptom onset to diagnosis was 14.5 months (SD = 30.7). 

The radiological findings demonstrated a range of complications associated with shoulder prosthesis, observed primarily through plain X-rays (eight cases; 80% [25,27,28,29,30,31,32,34]) and computer tomography imaging (one case; 10% [26]). In particular, in six cases (60% [27,28,29,30,31,32]), radiographic lucencies or loosening of the humeral stem were observed. Furthermore, one case (10% [30]) presented with a dislocation of a shoulder prosthesis, accompanied by loosening signs located at the proximal metaphysis of the humerus and a fracture of the greater tuberosity, while another (10% [29]) had dislodgement of the glenoid component of the prosthesis. Finally, in two cases, the X-rays were without evidence (20%) [25,34]) (Table 3).

Regarding the laboratory examinations, the white blood cells (WBC) were reported in three cases (30% [25,29,30]), with mean value 18.28 cells/μL (SD = 13.65); the c-reactive protein, (CRP) was reported in six cases (60% [25,27,28,30,31,34]), with mean value 27.43 mg/L (SD = 19.43); and the erythrocyte sedimentation rate (ESR) was reported in six cases (60% [25,27,28,29,31,34]), with mean value 54 mm/h (SD = 31.82 mm/h).

Analyzing the number of antifungal agents used, it was observed that a single antifungal agent was used in three cases (30% [28,30,32]), while two antifungal agents, either simultaneously or consecutively, were used in another four (40% [25,29,31,34]). There was also one case (10% [27]) where more than two antifungal agents were given. In two cases (20% [26,33]), the data regarding the specific antifungal agent were not provided. The mean duration of AFT was 8.4 weeks (SD = 4.8).

Fluconazole was the preferred agent in six cases (60% [25,27,28,30,31,34]), in two (33.3% [28,30]) as monotherapy, followed by amphotericin B in three cases (30% [29,31,32]), in one (33.3% [32]) as monotherapy, and micafungin in two patients (20% [25,27]) as combination therapy. Caspofungin, ketoconazole, and voriconazole were administered in one case (10% [27,29,34]) each, as part of combination therapy. For the *Candida* spp. shoulder PJIs, the preferred AFT was fluconazole [five cases (55% [25,27,28,30,34]), in two (40% [28,30]) as monotherapy], followed by amphotericin B [two cases (22.2% [29,32]), in one (50% [32]) as monotherapy] and micafungin [two cases (22.2% [25,27]), none as monotherapy]. Furthermore, caspofungin, ketoconazole, and voriconazole were used in one case (11.1% [27,29,34]) each (none as monotherapy). In the single infection [31] caused by *Cryptococcus magnus*, the treatment included the combination of amphotericin B and fluconazole.

Regarding surgical management of these infections, a resection arthroplasty (RA) was performed in most of the reported patients (five cases; 50% [27,29,30,31,32]), followed by two-stage revision arthroplasty (2-SRA) (three cases; 30% [26,28,34]) and one-stage revision arthroplasty (1-SRA) (one case; 10% [33]), while in one case (10% [25]), surgical debridement with implant retention was performed. Regarding the RA cases, in one case [31] that was initially planned for 2-SRA, the patient refused to undergo the second stage, and long-term retention of the spacer was decided. Cement spacers were used in four cases (40% [26,27,28,31]), and in all cases an antibiotic regimen was impregnated.

Infection’s outcome was successful in nine cases (90%) [25,26,27,28,29,30,31,32,34]), while in one case the outcome was not reported [33].

## 4. Discussion

PJIs are a severe complication following prosthetic joint reconstruction surgery and one of the most common causes of revisions [35,36]. Fungi as causative organisms of PJIs are considered rare. Nevertheless, their incidence has increased over the last few years, making fungal PJIs a challenging infection with high comorbidity. Fungal PJIs have been mostly studied in knee and hip arthroplasties [37,38,39]. Although there are no official guidelines for treating fungal PJIs, current recommendations in knee and hip arthroplasty, based on limited evidence, advocate for a two-stage revision arthroplasty (2-SRA) along with prolonged antifungal treatment (AFT) [18,22]. These infections have not been studied thoroughly in SA; hence, treatment is based on data from hip and knee infected prostheses. 

The present systematic review analyzed all reported cases, so far, of shoulder fungal PJI. This review included cases of anatomic and reverse SA, as well as of hemiarthroplasty. The demographics, the clinical and laboratory findings, the microbiological examination findings, and the diagnostic and therapeutic approach of each case were evaluated in an effort to better understand these rare infections.

Complication rates following SA vary between 7% and 25%. Among these complications, PJIs are estimated to occur in 3% to 8% of cases and can result in increased healthcare costs that are estimated to be more than double those of the initial procedure [40,41]. The incidence of PJI for primary SA ranges between 0.4% and 1.9% [42,43,44]. To further minimize the risk of these infections, comprehensive bundles of preventive measures have been proposed [36,45,46]. It should be noted that in revision SA, this incidence climbs up to 3.9% [42,43,44]. Of the present cases, one could be characterized as revision SA, since the patient had previously undergone internal fixation for a proximal humerus fracture. An important issue that should be mentioned is the late diagnosis of these infections. The mean time-interval from symptoms’ onset to diagnosis was about 14.5 months. Shoulder PJIs caused by fungi seem to be indolent infections; hence, the characteristic signs may be absent or not so evident. Additionally, the hematogenous spread in most cases (30%) may also play a role in the late diagnosis. 

The most common causative fungus was *Candida* spp. in this systematic review. The infections of a prosthetic joint from *Candida* lead to the formation of biofilms on the surface of the prosthesis, contributing to the persistence of the infection. Biofilms protect the fungal cells from antifungal agents and the host immune response, making the infection more difficult to treat than planktonic (free-floating) organisms. *Candida* spp. are opportunistic fungi that can colonize the skin and mucosal surfaces, particularly in immunocompromised individuals or those with underlying chronic conditions such as DM. It is notable that in the present population sample, 30% suffered DM [25,27,31], while 70% were immunocompromised [25,26,27,29,30,31,34]. DM holds substantial clinical relevance due to its detrimental effects on both innate and adaptive immune responses, which increase susceptibility to fungal infections [47,48]. DM is associated with notable impairments in neutrophil functions, including reduced phagocytosis, compromised chemotaxis, and diminished cytokine production. Additionally, the chronic hyperglycemia characteristic of DM promotes a shift towards a Th2-dominant immune response, which adversely affects Th1-dependent immunity. This change further compromises the host’s capacity to organize an effective defense against fungal pathogens [47,48].

Another potential risk for fungal infections is previous prolonged antibiotic treatment. This risk factor results in invasive fungal infections, including *Candida* species, and it disrupts the ecology of the normal microbial flora of the host [49,50]. In the present sample, 50% had received some antibiotic before the fungal infection. Careful use of antibiotic is of utmost importance for the avoidance of future invasive fungal infections and, of course, pathogens’ resistance [51]. Fungal PJIs in the hip and knee, caused by *Candida* spp., account for over 85% of cases [38]. In the current review, *Candida* spp.-associated fungal PJIs of the shoulder were reported in 90% of cases [25,26,27,28,29,30,32,33,34].

Non-*Candida* PJIs represent much rarer infections, on which even fewer data exist. In a recent review, a total of 42 cases of non-*Candida* fungal PJIs were evaluated, with *Aspergillus* being the most common isolated fungus [20]. In the present sample, a case of *Cryptococcus magnus* was identified. While *Cryptococcus magnus* infections are rare, they can present as serious opportunistic infections, especially in patients with compromised immune systems, such as those with HIV/AIDS, organ transplant recipients, or individuals undergoing immunosuppressive therapy. Infection with *Cryptococcus magnus* typically manifests as cryptococcosis, which can affect the lungs and central nervous system or disseminate to other organs. The pathogen’s ability to form a polysaccharide capsule contributes to its virulence, aiding in immune evasion and persistence within the host [52].

Co-infection with bacterial pathogens was observed in four cases (40%), with MRSE, *Serratia marcescens*, and *Staphylococcus* species being identified, while a fourth case had another site of infection, in the lungs [32]. It is noteworthy that bacterial co-infection has been reported in approximately 15% to 20% of PJI cases caused by fungi [53]. It is believed that bacteria and fungi work together within the prosthetic biofilm, leading to more aggressive infections [54]. Patients with more than two co-infective and, especially, multidrug-resistant organisms, face a heightened risk of recurrent infection, which should be taken into account when determining treatment and prognosis [20,54].

Definite diagnosis of a fungal PJI may be established through microbiological, pathological, or serological examination. In this review, fungal species were cultured in all of the cases presented. Regarding the sample that was cultured, the tissue samples (60%) were the most used, followed by joint fluid aspiration (40%). Routine blood culture systems, both manual and automated, generally support the growth of yeasts like *Candida* species; however, they may miss molds, especially dimorphic fungi. If fungal infection is suspected and cultures are negative, alternative methods like lysis centrifugation or advanced techniques (e.g., MALDI-TOF, PCR) should be considered [55]. Accurate species identification is crucial for appropriate treatment, though some of these methods are not widely available or fully validated [55]. Cultures should be observed for four weeks before concluding that they are negative for growth [56]. A study showed that fungi were detected only after examining scrapings from the surface of the extracted prosthesis [57]. Other research has confirmed the presence of fungal prosthetic infections by identifying fungi directly in bone specimens [48,58]. It is crucial to emphasize the importance of repeating fluid cultures and obtaining multiple positive tissue cultures before diagnosing fungal periprosthetic joint infections [59,60,61].

In PJIs, the initial diagnostic algorithm includes clinical examination, plain radiography, and measurement of serum inflammatory markers (e.g., ESR and CRP) [62,63]. In the present review, local symptoms, including pain and high local temperature, were evident in most cases, while fever was present in about 20%. Furthermore, inflammatory markers were found to be elevated. In particular, the mean WBC was 18.28 cells/μL, CRP 27.4 mg/L, and ESR 54 mm/h. Nevertheless, these symptoms, signs, and laboratory examinations do not differentiate fungal from bacterial PJIs. A high clinical suspicion should be present, especially in immunocompromised patients.

Plain X-rays may exhibit signs of prosthesis loosening that are not specific for infection. These findings should always be interpreted in correlation with the clinical and laboratory findings of the patient [64]. In this population sample, 60% of patients had signs of loosening in radiographs.

Based on this review’s findings, a combination of lengthy AFT and surgical intervention seems to be the standard of care. In particular, in the present patient sample, the mean duration of AFT was 8.41 weeks. Fluconazole and amphotericin B were the preferred agents either as monotherapy or in combination with other drugs. Fluconazole has infrequently been linked to severe hepatotoxicity [65]. Consequently, it is advisable to monitor liver function tests regularly during extended courses of fluconazole therapy. On the other hand, while amphotericin B remains a potent broad-spectrum antifungal agent, its use is often limited by its toxicity, particularly renal dysfunction, which poses a challenge for the prolonged treatment necessary for managing PJIs [66]. Although liposomal formulations of amphotericin B have significantly mitigated its nephrotoxic effects, the potential for long-term complications still exists with these formulations. More recently, continuous local antibiotic perfusion has been used in fungal PJIs. The local continuous antibiotic perfusion can deliver high concentrations of antimicrobial or antifungal agents directly to the affected area. It has been used in the past for osteosynthesis cases in fractures [67]. This technique allows for the delivery of high concentrations of antimicrobials at low flow rates, effectively targeting bone marrow through intra-medullary antibiotic perfusion, soft tissues via intra-soft-tissue antibiotic perfusion, and joints with intra-joint antibiotic perfusion. Additionally, it concurrently removes synovial fluid, hematomas, and leftover antimicrobials by employing a combination of negative pressure wound therapy and drainage tubes. Consequently, this method may be beneficial in treating fungal prosthetic joint infections by delivering effective antifungal agents directly to the local infection site while managing dead spaces [67].

Regarding surgical intervention, most cases were treated with RA (50%). Multiple revision surgeries of SA are extremely challenging, and major osseous defects may prohibit the implantation of a new prosthesis. Furthermore, fungal PJIs are very difficult-to-treat infections, in the majority of cases requiring implant removal. Utilizing a permanent cement spacer may represent a dependable approach for managing PJI in elderly, low-demand patients with comorbid conditions, where minimizing the need for additional surgical interventions is preferred [68]. The use of antifungal agents in the spacer could also prove beneficial, and further research is warranted [69,70]. In the present patient sample, no antifungal regimen was used during the mixing of the antibiotic-loaded cement spacer. Additional factors, such as humeral stem loosening or joint instability (dislocation), may also influence the decision-making process for appropriate surgical intervention. In cases of prosthetic loosening, replacement should be performed in either one or two stages, and the underlying cause of the dislocation must be thoroughly investigated prior to surgery.

A 2-SRA was performed in three cases from the present sample. A recent review, comparing 1-SRA and 2-SRA for shoulder PJI, showed that 1-SRA was more successful in the eradication of infections and exhibited fewer complications [43,71]. Nonetheless, due to the limited patient sample sizes, the generally low quality of the available studies, and insufficient data on clinical severity and bacterial virulence, caution should be exercised when interpreting these findings [43]. Furthermore, no data on fungal infections are provided in this context. Taking into account 1-SRA for hip and knee PJIs, the isolation of fungus seems to be a contraindication for performing 1-SRA [72,73].

This systematic review faces several limitations that must be acknowledged. A significant constraint is the incomplete availability of detailed information from the published cases. For instance, critical data such as dosages, drug serum levels, minimum inhibitory concentrations (MICs), and side effects of the antifungal medications were frequently omitted. Additionally, the review is based solely on case reports, without a specific classification system for the diagnosis, with no cohort studies with extended follow-up available to provide a broader perspective or enhance the robustness of the evidence. Despite these limitations, this review provides a valuable foundation for future clinical reference and research. Reporting and analyzing fungal PJIs in the shoulder are crucial for advancing our understanding of diagnostic and therapeutic approaches to them.

## 5. Conclusions

The diagnosis and management of fungal periprosthetic PJIs in the shoulder present significant challenges, often exceeding those encountered in total hip or knee arthroplasties. These rare but severe infections necessitate a multidisciplinary approach, as the combination of AFT and tailored surgical strategies appears to be the most effective treatment. The surgical management of shoulder PJIs can be particularly challenging due to the potential for substantial bone defects, underscoring the importance of meticulous preoperative planning. Given the inconclusive outcomes of current treatment protocols, there is a critical need for further research to refine and establish optimal management strategies. Additionally, the unique considerations for shoulder PJIs, such as the potential use of permanent spacers in low-demand patients or RA, highlight the differences in treatment approaches compared to hip and knee infections. Continued investigation and reporting of these fungal infections will enhance our understanding and improve diagnostic and therapeutic approaches for this complex condition.

## Figures and Tables

**Table 1:** Quality assessment of case report studies using Murad et al. tool [24].

Study	D1	D2	D3	D4	D5	D6	D7	D8	Overall Risk of Bias
Lo, E.Y. et al. [26]	Y	Y	Y				Y	Y	Low
Baptista, M. et al. [31]	N	Y	Y				Y	Y	Moderate
Luengo-Alonso, G. et al. [28]	N	Y	Y				Y	Y	Moderate
Skedros, J.G. et al. [27]	N	Y	Y				Y	Y	Moderate
Radha, S. et al. [30]	N	Y	Y				Y	Y	Moderate
Grosso, M.J. et al. [33]	Y	Y	Y				Y	Y	Low
Jason Springer et al. [25]	N	Y	Y				Y	Y	Moderate
Antony, S. et al. [34]	N	Y	Y				Y	Y	Moderate
Lim, E.V. et al. [32]	N	Y	Y				Y	Y	Moderate
Lichtman, E.A. et al. [29]	N	Y	Y				Y	Y	Moderate

D1. Do(es) the patient(s) represent the whole experience of the investigator (center), or is the selection method unclear to the extent that other patients with similar presentation may not have been reported? D2. Was the exposure adequately ascertained? D3. Was the outcome adequately ascertained? D4. Were other alternative causes that may explain the observation ruled out? (not use) D5. Was there a challenge/rechallenge phenomenon? (not use) D6. Was there a dose–response effect? (not use) D7. Was follow-up long enough for outcomes to occur? D8. Is the case(s) described with sufficient details to allow other investigators to replicate the research or to allow practitioners to make inferences related to their own practice?

**Table 2:** Patients’ demographics, responsible fungus, bacterial co-infection, comorbidities.

Case No.	Author	Country of Origin	Year	Patients (Number)	Type of Arthroplasty	Gender/Age	Fungus Isolation	Other Sites of Infection (Prior or Concomitant)	Immunosuppressive Conditions	Type of Previous Antibiotic Therapy
1.	Lo, E.Y. et al. [26]	USA, SPAIN	2023	1	TSA	F/40	*Candida* species	NR	2 (not specified)	cephazolin, vancomycin
2.	Baptista, M. et al. [31]	Portugal	2020	1	TSA	M/68	*Cryptococcus magnus*	MRSE	DM, CHF, PE, COPD, AF	linezolid vancomycin, gentamicin
3.	Luengo-Alonso, G. et al. [28]	Spain	2018	1	RSA	M/64	*Candida parapsilosis*	NR	NR	NR
4.	Skedros, J.G. et al. [27]	USA	2014	1	RSA	M/58	*Candida glabrata*	*Serratia marcescens*	DM, CKD, AF	NR
5.	Radha, S. et al. [30]	UK	2012	1	RSA	F/71	*Candida albicans*	NR	RA	NR
6.	Grosso, M.J. et al. [33]	USA	2012	1	NR	NR/NR	*Candida parapsilosis*	NR	NR	NR
7.	Jason Springer et al. [25]	USA	2012	1	Hem	F/65	*Candida albicans*	NR	DM, RA	vancomycin, ceftriaxone
8.	Antony, S. et al. [34]	USA	2008	1	TSA	F/67	*Candida parapsilosis*	*Staphylococcus* species	depression, nicotine abuse	daptomycin
9.	Lim, E.V. et al. [32]	USA	1989	1	Hem	F/70	*Candida albicans*	pulmonary infection	NR	doxycycline
10.	Lichtman, E.A. et al. [29]	USA	1983	1	TSA	M/59	*Candida parapsilosis*	NR	drug abuse, alcoholism	NR

CHF: congestive heart failure, PE: pleural effusion, COPD: chronic obstructive pulmonary disease, AF: atrial fibrillation, CKD: chronic kidney disease, DM: diabetes mellitus, RA: rheumatoid arthritis, TSA: total shoulder arthroplasty (anatomic), RSA: reverse shoulder arthroplasty, Hem: hemiarthroplasty. Immunosuppression: previous antibiotics within a year and type. NR: not reported.

**Table 3:** Mechanisms of infection, clinical manifestations (symptoms), and microbiological and imaging findings are presented.

Case	Mechanism of Infection	Clinical Manifestations	Microbiological Examination	Concomitant Bacteria	Radiological Findings	Type of Imaging Studies (X-ray, US, MRI, CT Scan)
1.	NR	NR	tissue specimen	NR	NR	CT
2.	hematogenous	lack of clinical sign	tissue specimen	MRSE	stem loosening	Ro
3.	surgical site infection	fever, pain, warmth, swelling	tissue specimen	NR	stem loosening	Ro
4.	NR	pain, instability	joint fluid	*Serratia marcescens*	radiographic lucencies	Ro
5.	hematogenous	swelling, erythema, warmth, motion limitation	tissue specimen	NR	dislocated shoulder prosthesis, loosening around the proximal humeral metaphysis, and a fractured greater tuberosity	Ro
6.	NR	NR	tissue specimen	NR	NR	NR
7.	hematogenous	warmth, swelling, pain, motion limitation	joint fluid	NR	without evidence	Ro
8.	NR	pain, swelling, erythema	joint fluid	*Staphylococcus* species	without evidence	Ro
9.	NR	fever, swelling, pain	tissue specimen	NR	stem loosening	Ro
10.	NR	motion limitation, swelling	joint fluid	NR	stem loosening and dislodgement of the glenoid component of the prosthesis	Ro

CT: computer tomography, NR: not reported, Ro: radiographs, MRSE: methicillin-resistant *Staphylococcus epidermitis*.

## Data Availability

All data are available upon reasonable request to the corresponding author.
